# FINANCIAL EXPLOITATION OF OLDER ADULTS: INTERDISCIPLINARY PERSPECTIVES ON NEW DIRECTIONS

**DOI:** 10.1093/geroni/igad104.0880

**Published:** 2023-12-21

**Authors:** Julie Miller

## Abstract

Financial exploitation of older adults is a topic that has drawn increased public recognition in recent years. Despite advances in research, services, products, and policies working to prevent financial exploitation of older adults and support victims, interdisciplinary perspectives are needed to strengthen interventions. This symposium will bring together scholars and practitioners to address the issue of financial exploitation from a variety of disciplines, all united in their mission to detect and prevent financial exploitation of older adults and to support victims. The first two presentations in the symposium will highlight conceptual understandings of financial exploitation of older adults, including a revisionary model of financial exploitation of older adults that includes emerging cognitive, cultural, and other contextual factors (Presentation 1) and a study of financial vulnerability and mental health as they are associated with financial exploitation, particularly when perpetrated by trusted others (Presentation 2). The remaining presentations will describe research from cross-sector, cross-industry, interventions. Presentation 3 will describe study findings about a helpline for concerned persons of exploitation victims that dually offers services directed to primary victims of financial exploitation. Presentation 4 will highlight a study of case characteristics associated with a new adult protection law designed to protect older and vulnerable adults experiencing exploitation. Presentation 5 will describe a study of financial professionals’ experiences with, and attitudes toward, financial exploitation of aging clients. Findings from all five presentations will point to next steps for research and intervention across multiple disciplines, including social work, law and policy, healthcare, and financial services.

